# Valorization of Flaxseed Oil Cake Residual from Cold-Press Oil Production as a Material for Preparation of Spray-Dried Functional Powders for Food Applications as Emulsion Stabilizers

**DOI:** 10.3390/biom10010153

**Published:** 2020-01-17

**Authors:** Emilia Drozłowska, Łukasz Łopusiewicz, Monika Mężyńska, Artur Bartkowiak

**Affiliations:** Center of Bioimmobilisation and Innovative Packaging Materials, Faculty of Food Sciences and Fisheries, West Pomeranian University of Technology Szczecin, Janickiego 35, 71-270 Szczecin, Poland; emilia_drozlowska@zut.edu.pl (E.D.); mmezynska@zut.edu.pl (M.M.); artur-bartkowiak@zut.edu.pl (A.B.)

**Keywords:** agricultural residues, flaxseed, oil cake, spray drying, emulsifying properties

## Abstract

Flaxseed oil cake extract (residual from cold-press oil production and rich in proteins and polysaccharides) was evaluated as a potential substrate for the preparation of spray-dried powders with emulsifying activity. Three variants of powders were obtained using different spray-drying process inlet temperatures: 160 °C, 180 °C, and 200 °C. The influence of temperature on physicochemical features (water holding capacity, oil binding capacity, water activity, solubility, color, chemical composition, antioxidant activity, and surface morphology) of the powders was estimated. Additionally, the emulsifying activity of the powders and the stability of oil-in-water emulsions prepared with their various content (0.5%, 1%, and 3%) were determined. Results showed that inlet temperature had significant influence on all physicochemical and functional properties of the powders. Increased inlet temperature decreased solubility and antioxidant activity but increased water-holding capacity, oil-binding capacity, and emulsifying activity. The emulsions prepared with the powder obtained at 200 °C showed the highest stability. SEM images showed the production of relatively spherical particles which were folded or wrinkled with a lot of dentures. This study could open a promising pathway for producing natural and plant-based spray-dried powders for food applications as emulsion stabilizers.

## 1. Introduction

Annually, a major part of agricultural products is converted into by-products and waste material during processing [1]. Most of these ingredients are discarded or consumed as the feed for livestock [1,2]. Many of these by-products are rich in bioactive compounds that can be potentially applied on novel food product development (food additives and functional foods) through waste valorisation under the influence of market trends connected with zero waste and circular economy [1,2,3,4,5,6]. Increasing consumer awareness of health issues has created a fast-growing demand for plant-based food products [2]. Plant-based products are perceived as healthy foods, containing high levels of antioxidants, vitamins, dietary fibers, and minerals. One of the directions of application of plants is their use for the production of “plant milks”, which are colloidal suspensions or emulsions consisting of dissolved and disintegrated plant material (legumes, oilseeds, cereals, or pseudocereals) that resemble cow’s milk in appearance and are rich in proteins and other bioactive compounds [7]. There are many plant-based products available on the market such as soy, almond, oat, coconut, hemp, or rice milks [7,8]. Industries are forced to explore different raw materials or to use new technologies/food processes with the aim to develop new functional products. Additionally, increased utilization of plant proteins in human diet will be necessary to meet the nutritional requirements of the growing world population [9,10]. Current protein demand for the 7.3 billion inhabitants of the world is approximately 202 million tonnes globally [9]. UN figures projecting global population growth to 9.7 billion by 2050 seem to be generally accepted [9,11]. Moreover, nowadays, vegetal sources of protein dominate protein supply globally (57%), with meat (18%), dairy (10%), fish and shellfish (6%), and other animal products (9%) making up the remainder [9]. Plants other than soya have not been given enough attention not because they lack a protein that may be functionally or nutritionally equal to soybean but rather because of economic reasons. However, the future situation may change and it seems advisable at present to explore the development and utilization of a food variety, derived from or containing a wide range of plant proteins [2,10,12]. It is anticipated that, in the foreseeable future, technological development will continue to position plant-based protein as a desirable option from a sustainability perspective [9]. From a nutritional standpoint, with the right combination, plant proteins can supply sufficient amounts of essential amino acids for human health requirements [10]. Proteins are natural amphiphilic molecules with interfacial activity and colloid-stabilizing characteristics; hence, proteins are increasingly used as emulsifiers [10,13]. Proteins preferentially adsorb to the oil–water interface and form a viscoelastic film, which provides physical stability to the emulsion during subsequent processing and storage [14,15]. Plant proteins are increasingly used in various food applications due to their acceptable emulsifying, fat- and water-absorbing, texture-modifying, and whipping properties [12,15,16,17]. One of the interesting directions of applications of plant milks (due to their high content of proteins) could be their spray drying, which allows obtaining products with a wide spectrum of applications. Oil cakes/oil meals are by-products obtained after oil extraction from oilseeds [2,18,19]. There are two types of cakes: edible and nonedible. Edible cakes have a high nutritional value, i.e., their protein content ranges from 15% to 50%. Their composition varies depending on their variety, growing conditions, and extraction methods. They have been widely used for production of industrial enzymes, antibiotics, biopesticides, vitamins, and other biochemicals. Due to their rich protein content, they are used as an animal feed supplement, especially for ruminants and fishes [18].

Flaxseed oil cake (FOC) is a cheap by-product of flaxseed (*Linum usitatissimum* L.) oil pressing and is rich in omega-3 fatty acids, proteins, soluble and insoluble fibers, phytoestrogenic lignans, different types of antioxidant compounds, vitamins (A, C, D, and E), and minerals (Mg, K, Na, Fe, Cu, Mn, and Zn) [1,20,21]. Many works have reported positive influence of flaxseed consumption with possible applications in medical fields such as colon cancer prevention and reduction of the risk of cardiovascular disease [18,22,23,24]. It is considered a “superfood” and generally recognized as safe (GRAS). Altogether, flaxseed is an excellent plant food candidate that meets the needs of 21st century consumers in terms of being rich in nutrients as well as bioactive and functional ingredients [1,18,25,26,27,28]. However, some consideration in the application of various forms of flaxseed in human foods is related to the presence of antinutritional cyanogenic glycosides compounds (such as linustatin, neolinustatin, linamarin, and lotaustralin), which can be significantly reduced by various processes such as thermal, microbial, or enzymatic treatment [25,29,30,31,32]. In a previous study, it was demonstrated that, after thermal treatment, FOC is a safe raw material for food processing [2]. Flaxseed seeds contain 10 to 30% protein, which is mainly composed of amino acids such as glutamic acid, arginine, valine, leucine, tyrosine, and phenylalanine [24,33,34]. Flaxseed proteins are composed of salt-soluble 11–12S globulins and water-soluble 1.6–2S albumins, which are referred to as linin and conlinin, respectively. Flaxseed globulin has an overall molecular mass of ~320 kDa, a pI (isoelectric point) of ~4.75, and is comprised of at least five subunits having molecular masses ranging from 11 to 61 kDa held together by disulfide linkages [31,35]. In contrast, flaxseed albumin is a basic protein containing a single polypeptide chain that has a molecular mass between 16 and 18 kDa. Other important compounds of flaxseed seeds are polysaccharides commonly referred to as mucilage, which have nutritional value as a dietary soluble fiber which comprises about 8% of the seed weight. Flaxseed proteins have been investigated for their emulsifying properties [12,13,21,28,34,35]. From the standpoint of specific functional requirements, the preparation of FOC extracts containing both proteins and polysaccharides may be more desirable than the rejection of mucilage by expensive processes, since both protein and mucilage can be utilized in mixtures [13,20,28,35,36,37]. Martínez-Flores et al. reported that flaxseed protein with fiber (mucilage) obtained at pH 6 had a good emulsifying activity [38]. Also in the previous study, it was reported that FOC extract can be applied for production of low-fat mayonnaises due to its good emulsifying properties [37]. These features have led flaxseed and its by-products to be used as a rich source of functional ingredients for enriching and fortifying a variety of food products such as bread, dairy and nondairy products, juices, pasta, and meat products [1,2,18,27].

Vacuum freeze drying, spray drying, hot air drying, and atmospheric freeze drying are common drying methods for dehydration of food materials [1,29,33,39,40,41]. Spray drying is defined as a transformation of feed from a fluid state to dried particles by spraying the feed into a warm drying agent (usually air). Four main processing steps are involved in spray drying: atomization, mixing of hot gas and atomized particles, droplets evaporation, and collection of the dried products [40]. The final product is either powder, granular material, or agglomerate [39]. Some tensions in the spray-drying process such as the shear stress during atomization of the feed cause exposure of peptides and proteins at the air–water interface, increased temperature, denaturation, and aggregation, which can influence physiological and functional properties of peptides and proteins [1,16,40,42]. Given that different proteins have different sensitivities to drying-related stresses, it is of practical importance to investigate the effect of spray-drying methods on their properties and to relate these changes to the surface morphology of the dried product, hydrophobicity, and emulsifying properties.

To the best of our knowledge, there have been no reports about utilization of flaxseed oil cake obtained via cold-press technique to produce spray-dried extract. Thus, the aim of the presented study was to produce spray-dried powders from FOC extract and to examine the influence of inlet temperature on their physicochemical and emulsifying properties. The spray-dried powders reported in this study are novel in the functional food additives sector as final, ready to use products, and their evaluation in practical contribution as emulsifiers has not been previously reported in a scientific way.

## 2. Materials and Methods

### 2.1. Materials

Flaxseed oil cake (FOC) obtained via cold-press technique was kindly donated by ACS Sp. z o.o. (Bydgoszcz, Poland), and rapeseed oil (Zakłady Tłuszczowe, Kruszwica, Poland) was also used. According to the manufacturer’s information, the proximate composition of FOC was as follows: solid content 80.50%, ash content 4.50%, protein content 41.97%, fat content 6.11%, carbohydrates 27.99%, and fiber 6.29%. Sodium dodecyl sulphate (SDS), Sudan III, sodium hydroxide, Benedict’s reagent, phenol solution (5%), sulfuric acid (96%), glucose, bovine serum albumin, Ellman’s reagent (5,5′-dithiobis-2-nitrobenzoic acid), β-mercaptoethanol, trichloroacetic acid, urea, glycine, ethylenediaminetetraacetic acid (EDTA), tris(hydroxymethyl)aminomethane (Tris), ethanol, 2,2-diphenyl-1-picrylhydrazyl (DPPH), 2,2′-azino-bis(3-ethylbenzothiazoline-6-sulfonic acid) (ABTS) and methanol were purchased from Sigma Aldrich (Darmstadt, Germany). All reagents were of analytical grade.

### 2.2. Preparation of Flaxseed Oil Cake Extract (FOCE)

The preparation process of FOCE (Flaxseed Oil Cake Extract) consisted of a few steps. At the beginning, the FOC was mixed with distilled water in a ratio 1:10 (*w*/*w*). Then, the mixture was heated at 90 °C for 1 h with constant stirring (250 rpm) After this, the mixture was cooled down to room temperature and centrifuged (2500 rpm) for 30 min at 20 °C (MPW-352R, MED Instruments, Warsaw, Poland). The supernatant was filtered under vacuum to obtain a clear, milky fluid. FOCE was homogenized for 5 min with a homogenizer (SilentCrusherM, Heidolph, Germany) at 12,000 rpm. The protein content was determined by the micro biuret method [43], whereas saccharide content was determined based on the phenol-sulfuric acid reaction [44]. All spectrophotometric measurements were performed on Thermo Scientific Evolution 220 spectrophotometer (Thermo Fisher Scientific Inc., Waltham, DE, USA).

### 2.3. Preparation of Spray-Dried FOCE Powders

Spray-drying of FOCE was carried out using a lab-scale spray dryer (Büchi B-290, Büchi Labortechnik AGT, Flawill, Switzerland). Three inlet temperatures, 160 °C (powder A), 180 °C (powder B), and 200 °C (powder C), were used. The outlet temperature was maintained at 60 ± 5 °C during the spray-drying process. The flow rate of the FOC was maintained at 6.5 mL/min by keeping the pump capacity at 20%.

### 2.4. Determination of Total Solids Content, Solubility, and Water Activity of Spray-Dried FOCE Powders

Total solid content of each powder was evaluated according to the AOAC (Association of Official Agricultural Chemists) standard method (no. 968.11) [45]. For the purpose of evaluating the level of solubility, 1 g of particular powder (W_1_) was added to a test tube (W_0_). The tubes were filled with 10 mL of distilled water, and samples were centrifuged (2500 rpm) for 2 min. The supernatants were decanted, and tubes were dried for 24 h at 50 °C. After this process, the tubes’ weight was determined again (W_2_). The solubility was calculated according to Equation (1) [16]:

(1) $$Solubility\;\left(\%\right)=\frac{W_{2}-W_{0}}{W_{1}}\times 100$$

Water activity (a_w_) was measured at 25 °C using MS1 Set-aw (Novasina, Lachen, Switzerland) equipment. The powder samples (approx. 1 g) were placed in the device and left for 30 min to stabilize. Then, the a_w_ values were obtained. Each sample was measured in triplicate.

### 2.5. Determination of Water-Holding and Oil-Binding Capacities

Water-holding capacity (WHC) and oil-binding capacity (OBC) of powders were determined according to Gong et al. with a slight modification [16]. To determine WHC, 1 g of a particular powder (W_0_) was placed in Falcon tubes and weighed together with the tubes (W_1_). Then, 10 mL of distilled water was added to the samples and was vigorously vortexed for 10 s. After thorough wetting, the samples were allowed to stand at room temperature for 30 min and then centrifuged at 3000 rpm for 20 min. The supernatants were decanted, and the tubes containing the sediments were weighed (W_2_). The WHC was calculated according to Equation (2):

(2) $$WHC=\frac{W_{2}-W_{1}}{W_{0}}$$

To determine OBC, 1 g of particular powder (W_0_) was placed in a Falcon tubes and weighed together with the tubes (W_1_). Then, 10 mL of rapeseed oil was added to the samples and they were vigorously vortexed for 10 s. The next steps were identical as in WHC determination. The OBC was calculated according to Equation (3):

(3) $$OBC=\frac{W_{2}-W_{1}}{W_{0}}$$

### 2.6. SEM Observations

The surface morphology of the powders was observed and acquired using a scanning electron microscope (Vega 3 LMU, Tescan, Brno, Czech Republic). The samples were directly deposited on aluminium stubs using a double-sided adhesive carbon conductive tape and coated with a thin cold layer with the help of gold sputter. An accelerating potential of 15 kV was used during microscopic observation.

### 2.7. Determination of Sulfhydryl Groups (–SH) and Disulfide Bonds (–S–S–) Contents

The sulfhydryl groups (–SH) and the disulfide bonds (–S–S–) contents were determined following the methodology of Gong et al. [16]. Powder samples (180 mg) were mixed with 30 mL of Tris-glycine buffer (0.086 M Tris, 0.09 M glycine, 4nM EDTA, and pH 8.0) with 8 M urea for 30 min on the magnetic stirrer (150 rpm); then, the solutions were centrifuged at 6000 rpm for 10 min. After that, supernatants were collected for further analyses.

The –SH content was measured in 4 mL of supernatants, which were mixed with 160 µL of Ellman’s reagent (4 mg/mL), and absorbance of the mixtures was measured at 412 nm. To determine the -S-S- content, 8 µL of β-mercaptoethanol was added to 4 mL of supernatants. Mixtures were incubated at 25 °C for 2 h and after incubation, 10 mL of 12% trichloroacetic acid (TCA) was added. The mixtures were again kept at 25 °C for 1 h and centrifuged at 6000 rpm for 10 min. The precipitates were washed with 5 mL of TCA three times and dissolved in 6 mL of Tris-Glycine buffer. Ellman’s reagent was mixed with 4 mL of dissolved precipitates, and the absorbance of the samples was measured at 412 nm. The solution devoid of samples was used as a control. The content of –SH and –S–S– were calculated according to Equation (4):

(4) $$–SH\left(\mu \mathrm{mol}/g\right)=\frac{73.53\times A412}{C}$$

where A412 is absorbance at 412 nm; C is the sample concentration (mg of powder/mL); and Q_1_ and Q_2_ stand for the –SH contents before and after β-mercaptoethanol addition in the supernatants, respectively.

(5) $$–S–S–\left(\mu \mathrm{mol}/g\right)=\frac{Q1-Q2}{2}$$

### 2.8. FTIR Analysis of Powders

The FTIR spectra of the samples were obtained at room temperature by attenuated total reflection with an FTIR spectrometer (Perkin Elmer Spectrophotometer 100, Waltham, MA, USA). The samples (100 mg) were then scanned at a range between 650 cm^−1^ and 4000 cm^−1^ (100 scans and 4 cm^−1^ resolution). The obtained spectra were normalized, baseline corrected, and analyzed using SPECTRUM software (v10, Perkin Elmer Spectrophotometer, Waltham, MA, USA).

### 2.9. Determination of Powders Antioxidant Activity

The DPPH radical scavenging activity of powders was determined based on the method of Tong et al. with a slight modification [46]. One milliliter of dissolved powder (0.2 mg/mL) was mixed with 1 mL of 0.01 mM DPPH methanolic solution. The mixture was shaken vigorously and placed in darkness for 30 min. After the incubation, absorbance was measured at 517 nm and the DPPH inhibition ability was obtained from Equation (6), where Abs0 is absorbance without sample and Abs1 is absorbance in the presence of the sample:

(6) $$\%\;\mathrm{DPPH}\;\mathrm{inhibition}=⎡1-\frac{\left(\mathrm{Abs}0-\mathrm{Abs}1\right)}{\mathrm{Abs}0}⎤\times 100$$

Antioxidant activity of powders was also estimated by ABTS radical cation decolorization. Radical 2,2′-azino-bis(3-ethylbenzothiazoline)-6-sulphonic acid (ABTS^+.^) was produced by mixing 7 mM ABTS with 2.45 mM potassium persulfate (5 mL of ABTS + 5 mL of potassium persulphate 4.9 mM). The mixture was then incubated for 16 h in the dark at room temperature and subsequently diluted with water to an absorbance of maximum 1.00 at 734 nm. Sample A0 was the ABTS reagent used as a reference. The samples (50 µL) were mixed with 3 mL of diluted ABTS^+^. The absorbance was measured at 734 nm after 6 min of initial mixing (A1). The ABTS radical scavenging rate was calculated using Equation (7) [47], where Abs0 is absorbance without sample and Abs1 is absorbance in the presence of the sample.

(7) $$\%\;\mathrm{ABTS}\;\mathrm{inhibition}=⎡1-\frac{\left(\mathrm{Abs}0-\mathrm{Abs}1\right)}{\mathrm{Abs}0}⎤\times 100$$

### 2.10. Emulsifying Properties of Powders and Emulsions Characterization

Emulsions with 20% of the rapeseed oil phase were prepared by dissolving 0.5%, 1%, and 3% *v*/*v* of particular powder in distilled water and preliminarily mixing on a magnetic stirrer for 5 min (250 rpm); then, the emulsions were homogenized for 5 min at 15,000 rpm with a homogenizer (SilentCrusherM, Heldolph, Germany). Final emulsions were cold stored at 4 °C. To determine the emulsifying properties of powders, emulsion stability index (ESI) and emulsion activity index (EAI) were determined as described elsewhere [48,49]. Twenty µL of each emulsion was mixed with 5 mL of 0.1% SDS solution and vortexed; then, the absorbance was measured at 500 nm. The stability of emulsions was calculated according to Equation (8), where A_0_ is the initial absorbance (0 min) and A_10_ is the absorbance after 10 min.

(8) $$\mathrm{ESI}=\frac{A_{0}}{A_{0}-A_{10}}\times 100$$

Emulsion activity index (EAI) was calculated according to Equation (9):

(9) $$\mathrm{EAI}\;\left(m^{2}/g\right)=\frac{2\times 2.303\times A0\times \mathrm{DF}}{C\times 1\mathrm{cm}\times \mathrm{oil}\;\mathrm{fraction}\;\mathrm{volume}\times 10000}$$

where A_0_ is the initial absorbance (0 min), DF is the dilution factor (200), and C is the concentration of powder (g/mL).

### 2.11. Determination of Emulsions Particles Size Distribution

Particle size distribution measurements were performed using a Mastersizer 2000 (Malvern Instrument Ltd., Worcestershire, UK). Emulsions diluted with 0.1% SDS were gently stirred and dispersed in distilled water (stirrer speed—2000 rpm) until an obscuration rate of 10% was obtained. Optical properties of the sample were defined as follows: refractive index 1.500 and absorption 1.00. Droplet size measurements were reported as the volume-weighted mean diameter d_4,3_ = ∑n_i_d_4i_/∑n_i_d_i3_, where n_i_ is the number of droplets of diameter d_i_. Dispersion index (SPAN) was calculated according to Equation (10) [50], where d_10_, d_50+_, and d_90_ are the diameters at 10%, 50%, and 90% of cumulative volume, respectively:

(10) $$\mathrm{SPAN}=\frac{d_{90-}d_{50}}{d_{10}}$$

### 2.12. Emulsions Optical Microscopic Examination

Freshly prepared emulsions were mixed in ratio 1:2 with 0.1% SDS and Sudan III as oil dye. Emulsion samples were observed with a microscope (OptaTech, Warsaw, Poland) at a magnification of 10× at room temperature with a digital camera.

### 2.13. Powders and Emulsions Color Measurements

Powders and emulsions were measured for color by a Konica Minolta CR-5 colorimeter with the Hunter LAB color system (Konica Minolta, Osaka, Japan). Color coordinates were expressed as lightness (L*), redness/greenness (±a*), and yellowness/blueness (±b*).

### 2.14. Statistical Analysis

All experiments were replicated three times. Results are expressed as mean ± standard deviation. All data were subjected to a one-way analysis of variance (ANOVA) test using the software Statistica 13.0 (StatSoft, Kraków, Poland). Significant differences between means were determined by Fisher’s LSD (Least Significant Difference) NIR multiple comparison tests at *p* < 0.05.

## 3. Results and Discussion

### 3.1. The Proximate Composition of FOCE

The proximate composition of FOCE is listed in Table 1. It was noticed that FOCE had 3 ± 0.2% of dry matter and contained 14 ± 0.2 mg/mL of proteins, 6.5 ± 0.2 mg/mL of saccharides, and 9.5 ± 0.02 mg/mL of another extractable compounds.

### 3.2. The Changes of Water-Holding Capacity, Oil-Binding Capacity, Solubility, Dry Matter Content, Water Activity, Free Sulphydryl Groups, and Disulfide Bonds Contents

Water-holding capacity (WHC), oil-binding capacity (OBC), solubility, dry matter content, water activity (a_w_), as well as –SH (free sulphydryl groups) and -S-S- (disulfide bonds) contents of spray-dried FOCE powders are summarized in Table 2. For all samples, WHC and OBC higher than 100% were noticed. Similar values have been reported for spray-dried peanut protein isolate [16]. It was observed that, with increasing inlet temperature, the WHC and OBC of powders significantly increased (*p* < 0.05). Inlet temperature also had significant influence on solubility, dry matter content, and a_w_ of the powders (*p* < 0.05). It was noticed that the FOCE powder dried at 200 °C had the lowest solubility (64.82 ± 0.39%). Water affects the physical and textural characteristics of organic products as well as their chemical stability [39]. Also, it is known that the inlet temperature is one of the main factors determining moisture content in spray-dried products. In fact, the highest dry matter content as well as a_w_ were noticed for FOCE powder dried at 200 °C. As all the samples were characterized by water activity ranging from 0.52 ± 0.01 (160 °C) to 0.36 ± 0.00 (200 °C), it can be concluded that they would be an inappropriate environment for the development of microorganisms, as the recommended a_w_ value should be below 0.7 to avoid microbial growth in food products [39]. Very low water content has been reported for other spray-dried products [1,33,39]. It was observed that the inlet temperature influenced the –SH and –S–S– content of spray-dried FOCE powders, which are important functional groups and play substantial roles in functional properties of proteins [16]. As shown in Table 2, with increasing inlet temperature, the –SH content significantly decreased (*p* < 0.05), whereas –S–S– content significantly increased (*p* < 0.05). A similar observation was made by Gong et al. on spray-dried peanut protein isolate [16]. Sulfhydryl groups are part of the tertiary structure of proteins and participate in weak secondary bonds. All changes in the content of these groups is information about protein denaturation. Changes observed suggest that spray-drying caused partial denaturation of FOCE proteins, which also affects the functional characteristics of the proteins such as solubility [42].

### 3.3. The Changes of Powders Antioxidant Activity

As presented in Table 3, inlet temperature significantly influenced the antioxidant properties of FOCE powders (*p* < 0.05). With increasing inlet temperature, a decrease of DPPH as well as ABTS scavenging activities was noticed. A similar effect was observed for spray-dried soybean protein [51] as well as for spray-dried plant extracts [52]. Flaxseed is a rich source of phenolic compounds such as lignans (secoisolariciresinol diglycoside (SDG)) and phenolic acids [53]. SDG is a diphenolic that is conjugated with the mucilage and is water soluble. SDG and its aglycone (SECO) are the major lignans of flaxseed, showing antioxidant and efficient chemopreventive properties and having good thermal stability [22,54]. However, other antioxidant compounds may be less stable in high temperatures. Also spray drying is known to induce thermal denaturation and can change protein–protein hydrophobic, electrostatic, hydrogen-bonding, and disulfide-sulfhydryl interactions, which may affect the antioxidant activity of protein-rich extracts [16,33]. Nevertheless, despite the negative influence of inlet temperature, antioxidant activity of FOCE powder obtained in 200 °C remained at a relatively high level (69.23 ± 0.78% and 60.33 ± 0.30% for DPPH and ABTS, respectively) and is comparable to results reported for spray-dried egg white protein hydrolysate [33] and soybean protein [51]. The observed antioxidant activity of powders may be important considering their functionality in food formulations, such as emulsions based on oils with a high proportion of unsaturated fatty acids (such as linseed oil) that are sensitive to rancidity, which might determine their shelf life [55]. This can be particularly important in developing new products with a reduced content of fats and/or high level of unsaturated fatty acids [37].

### 3.4. The Changes in Powders Chemical Composition

**Figure 1:**
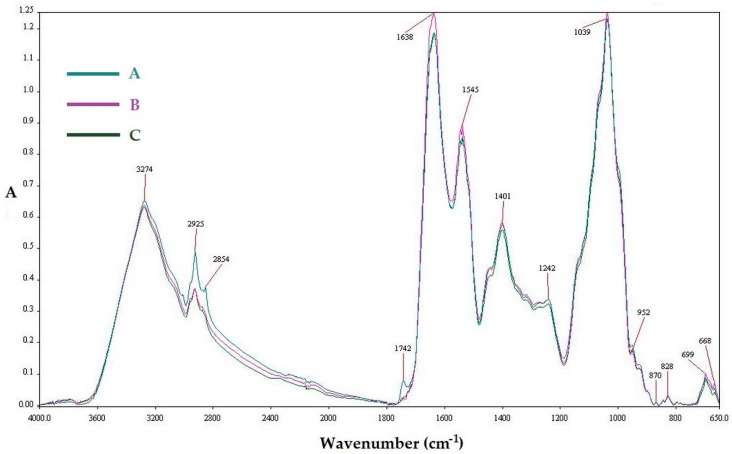
FTIR spectra of FOCE spray-dried powders; A—powder obtained at 160 °C; B—powder obtained at 180 °C; and C—powder obtained at 200 °C.

FTIR spectroscopy was used to investigate changes in the structure and chemical composition of FOCE powders caused by spray drying. Absorbance spectra are presented in Figure 1. As can be seen, the powders have characteristic CH_3_ and CH_2_ stretching peaks at approximately 2925 cm^−1^ and 2854 cm^−1^, respectively. At approximately 1638 cm^−1^, a strong amide I band was noticed which is attributed to C=O stretching vibrations, N–H stretching vibrations, N–H bending vibrations, and C–N bending vibrations in proteins [1,16]. In all spectra, a band at 3274 cm^−1^ was assigned to N–H stretching vibrations of the primary amide structure as well as O–H stretches, C–H stretches, and residual water [1,16]. A slight shift was observed with increasing inlet temperature, which can be due to N–H stretching coupled with hydrogen bonding [16]. Peaks recorded at 1039 cm^−1^ (angular deformation of =CH and =CH_2_ bonds), 828 cm^−1^ (deformation of C1-H and CH_2_), and 699 cm^−1^ (structural situation of the pyranose ring) can be linked with mucilage polysaccharides [1]. For the powder dried at 200 °C, an additional peak at 1742 cm^−1^ was observed, which may be assigned to the formation of hydrogen bonds between C=O and N–H groups of proteins with O–H of polyphenolics and polysaccharides [1,56].

### 3.5. The Change of Powders Color

Table 4 presents the color parameters of FOCE powders. Statistically significant differences in the color of powders were observed (*p* < 0.05). These results are partially in agreement with the study of Chen et al. [33], who noted that spray-drying caused reduction of L* and b* of egg white protein hydrolysates, and are partially in agreement with the results of Claussen et al., who observed lower L* and b* values but higher a* value of spray-dried potato protein concentrate [39]. The color of food products is important for their acceptability. A light color is expected to be more accepted by the customer. The reason might be that more applications exist when the product has additional brightness in it [39]. However, the color differences as a result of different inlet temperatures can be attributed to various content of cyanogenic compounds in flaxseed products. Oomah and Maza observed a lowering of the redness connected with reducing linustatin and neolinustatin content [29]. It is known that thermal treatment can reduce their content. It was also demonstrated in a previous study [2]. The color changes may be also linked with Maillard reactions [41] as well as the formation of polyphenolics–protein complexes [56].

### 3.6. Powders Surface Morphology

**Figure 2:**
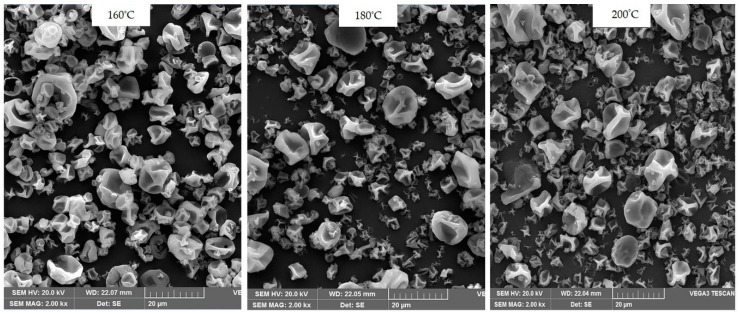
SEM images of FOCE spray-dried powders.

Figure 2 presents the surface morphology of spray-dried FOCE powders. SEM imaging showed the production of relatively spherical particles which were folded or wrinkled with a lot of dentures. This can be attributed to the interplay between rapidly decreasing diffusivity (i.e., rapidly increasing diffusivity in moisture removal) and increasing surface tension, which leads to the formation of collapsed particles with uneven/folded surface morphology. Polymeric macromolecules (such as proteins and carbohydrates) have very strong concentration-dependent moisture diffusivity, the magnitude of which decreases very rapidly when the solid content in droplet/particle increases [1,16]. In fact, it was observed that the powder obtained from the highest inlet temperature (200 °C) was characterized by the most uneven particle surface morphology. A higher proportion of particles with a larger diameter was also found in these powders. According to Chegini et al. [40], by increasing the inlet air temperature, a hard layer is formed on the particle top surface. This hard layer does not allow internal moisture to leave the particle and leads to its inflation, which increases the particle size. Similar surface morphologies have been reported for spray-dried flaxseed protein hydrolysates [1] and other protein rich products such as peanut protein isolate [16] and milk protein concentrate [42]. On the contrary, smooth surface of spray-dried mango powder has been reported by Caparino et al. [57].

### 3.7. Emulsifying Activity of Powders and Emulsions Stability

**Figure 3:**
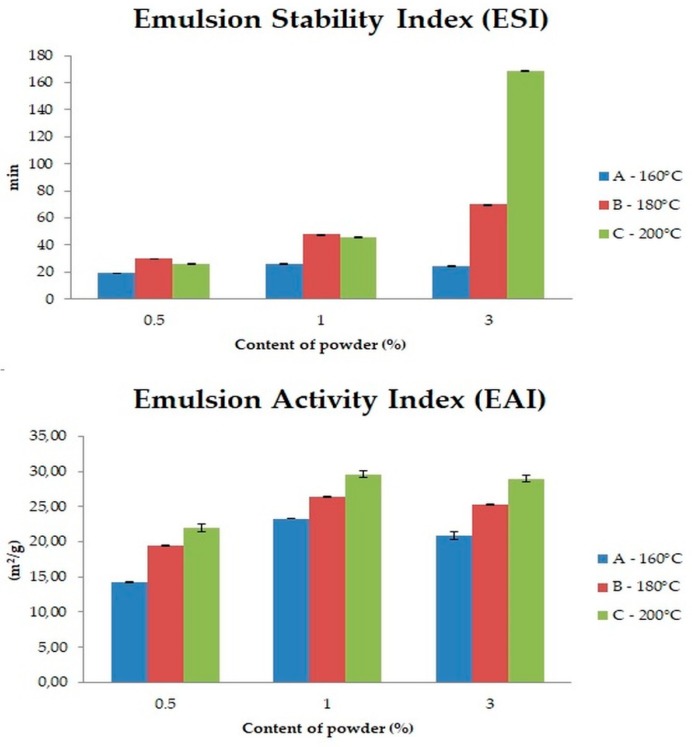
Emulsion Stability Index (ESI) and Emulsion Activity Index (EAI) of emulsions prepared with various content of FOCE powders; A—emulsions prepared with powder obtained at 160 °C; B—emulsions prepared with powder obtained at 180 °C; C—emulsions prepared with powder obtained at 200 °C; and 0.5%, 1%, and 3% are powder contents.

A thermal treatment processing may cause partial or complete denaturation of proteins, depending on the temperature level exposure time. The main process causing denaturation is the exposure to water of previously unexposed hydrophilic moieties and sulfhydryl groups. These moieties are responsible for the hydrophobic interactions with an oil phases, while the hydrophilic amino acid residues located on the surface may adsorb water [58]. With such a structure, which is a result of controlled heat treatment, FOC powders can interact with both the oil and water phases and can act as an effective emulsifying agent by stabilizing the phase interface, similar to some other heat-modified proteins [59,60]. The results of the presented study are comparable to results obtained by Molina et al. [61], who have shown a significant effect of thermal modification as specifying changes in the globulin fractions 7s and 11s, which are responsible for the emulsifying properties of this protein. According to Keerati-u-rai and Corredig [62], the application of heat in the modification of the soy protein, which is similar to flaxseed protein, leads to changes in intra-particle interactions. As shown in Figure 3, the inlet temperature had significant influence on emulsifying properties of the powders (*p* < 0.05), which was expressed by two stability indicators: EAI and ESI. EAI indicates the ability of an emulsifier to facilitate emulsion formation, and ESI indicates the ability to impart strength to the emulsion to resist destabilizing changes such as coalescence, creaming, flocculation, or sedimentation over a defined time period [12,16,63]. The highest ESI was described for emulsion C-3% (168.3 ± 0.01 min). Similarly, the highest EAI values were observed for emulsions prepared with the powder dried in 200 °C and are comparable with EAI described for canola protein isolate but slightly lower than EAI reported for flaxseed protein isolate [12].

**Figure 4:**
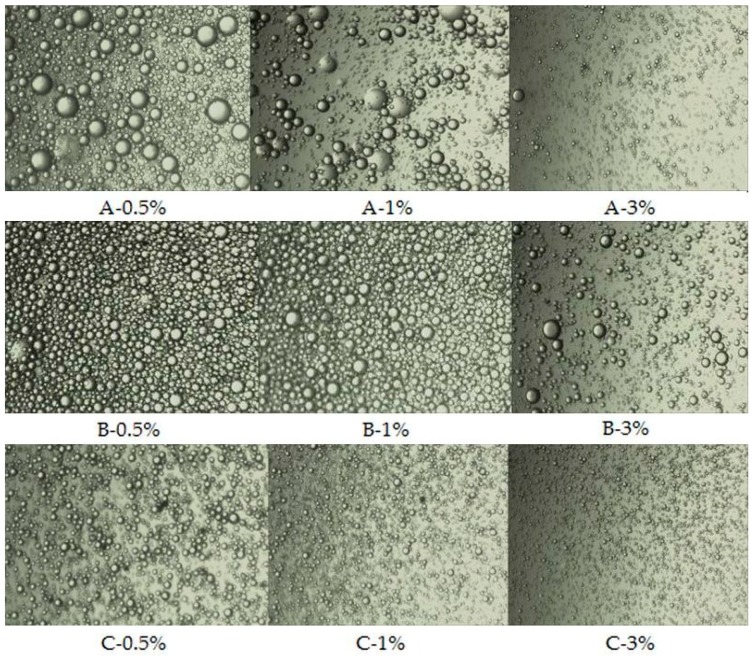
Optical microscopic images of emulsions prepared with FOCE powders obtained at various temperatures; A—emulsions prepared with powder obtained at 160 °C; B—emulsions prepared with powder obtained at 180 °C; C—emulsions prepared with powder obtained at 200 °C; and 0.5%, 1%, and 3%—powder contents.

Droplet size of emulsion always affects the stability of emulsions significantly. Emulsions with precisely controlled droplet size exhibit better stability [13]. Particle size (D_4_,_3_) is higher when fat in the sample is easily broken down and coalesced. Decrease of this parameter may indicate higher stability of emulsion. As shown in Table 5, the droplet size of the emulsions stabilised by powder dried at 200 °C was smaller than that of emulsions stabilised by powders dried at 180 °C and 160 °C, respectively (*p* < 0.05), which was in a good agreement with the decreased solubility and increased OBC of the powders with increased inlet temperature. Moreover, those results are in line with the results of microscopic observations presented in Figure 4. According to Pham et al. [56], it could be also attributed to formation of protein–phenolics complex. The authors reported that phenolic complexes of flaxseed protein isolate have better emulsifying activity than non-modified isolates. Similarly, von Staszewski et al. also reported that the stability of the oil-in-water emulsion stabilized by sunflower protein–chlorogenic acid complex was higher than the stability of emulsion stabilized by the sunflower protein [64].

SPAN gives information about how far are the emulsion particles diameters apart from the 10% of cumulative volume and 90% of cumulative volume points, normalized with the midpoint (50% of cumulative volume) [50]. Decreasing SPAN is the effect of better emulsion stability. As shown in Table 5, the lowest SPAN values were noticed for emulsions prepared by the use of the powder dried at 200 °C: C—0.5% (5.22), C—1% (4.39), and C—3% (3.71), which means that these emulsions were more homogenous than the emulsions prepared with powders dried at lower temperatures.

### 3.8. Emulsions Color

Results of the color determination could be useful for evaluation emulsion stability because these structures are very sensitive to color changes which are connected with aggregation. As can be seen in Table 6, the emulsions prepared with the powder dried at 200 °C exhibited significantly higher (*p* < 0.05) lightness (L*) values, which is a physical parameter referring to the ability of materials/products to reflect and scatter light [65]. This could be attributed to the much smaller oil droplet size of these emulsions samples as compared with the emulsions prepared with powders obtained at lower temperatures, which can scatter light more efficiently and which is in good agreement with particles size distribution measurements. It is well known that overall appearance of an emulsion is determined by a combination of light scattering and absorption phenomena which are responsible for the turbidity or lightness of an emulsion and color, respectively [66].

## 4. Conclusions

The results of this research indicated the potential of FOCE for production of spray-dried functional powders with different emulsifying activity. It was shown that inlet temperature has significant influence on both physicochemical features of powders and emulsions prepared with various contents. As the emulsions prepared with powder obtained at 200 °C were characterized by the highest stability and particles size distribution, it can be concluded that higher inlet temperature enhances functional properties of FOCE powders. This study could open a promising pathway for producing natural and plant-based spray-dried powders for food applications as emulsion stabilizers.

## Figures and Tables

**Table 1:** Dry matter, protein, saccharide, and other extractable compound content of Flaxseed Oil Cake Extract (FOCE).

Dry matter	3 ± 0.2%
Proteins	14 ± 0.2 mg/mL
Saccharides	6.5 ± 0.2 mg/mL
Other Extractable Compounds	9.5 ± 0.02 mg/mL

**Table 2:** Oil-binding capacity (OBC), water-holding capacity (WHC), solubility, dry matter, water activity (a_w_), and sulfhydryl group (–SH) and disulfide bond (–S–S–) contents of FOCE powders.

Powder Sample	OBC (%)	WHC (%)	Solubility (%)	Dry Matter (%)	a_w_	–SH (µmol/g)	–S–S– (µmol/g)
A	217.51 ± 0.40 ^c^	375.12 ± 0.30 ^c^	74.29 ± 0.24 ^a^	91.95 ± 5.06 ^a^	0.52 ± 0.01 ^a^	76.93 ± 0.05 ^a^	33.46 ± 0.02 ^c^
B	275.47 ± 0.25 ^b^	401.93 ± 0.31 ^b^	65.24 ± 0.57 ^b^	94.65 ± 0.86 ^a^	0.48 ± 0.00 ^b^	75.12 ± 0.01 ^b^	77.81 ± 0.14 ^b^
C	296.09 ± 0.12 ^a^	454.92 ± 0.27 ^a^	64.82 ± 0.39 ^b^	98.41 ± 2.75 ^a^	0.36 ± 0.00 ^c^	71.52 ± 0.00 ^c^	88.03 ± 0.08 ^a^

A—powder obtained at 160 °C; B—powder obtained at 180 °C; and C—powder obtained at 200 °C. Values are means ± standard deviation of triplicate determinations. Means with different letters in the same column are significantly different at *p* < 0.05.

**Table 3:** Antioxidant activity of FOCE powders.

Powder Sample	DPPH (%)	ABTS (%)
A	79.20 ± 0.16 ^a^	95.33 ± 1.01 ^a^
B	76.07 ± 0.78 ^b^	70.72 ± 1.30 ^b^
C	69.23 ± 0.78 ^c^	60.33 ± 0.30 ^c^

A—powder obtained at 160 °C; B—powder obtained at 180 °C; and C—powder obtained at 200 °C. Values are means ± standard deviation of triplicate determinations. Means with different letters in the same column are significantly different at *p* < 0.05. DPPH (2,2-diphenyl-1-picrylhydrazyl scavenging activity), ABTS (2,2′-azino-bis(3-ethylbenzothiazoline)-6-sulphonic acid scavenging activity).

**Table 4:** Color values of FOCE powders.

Powder Sample	L*	a*	b*
A	72.71 ± 0.01 ^c^	−1.06 ± 0.01 ^b^	17.74 ± 0.01 ^a^
B	72.74 ± 0.01 ^a^	−1.43 ± 0.01 ^a^	21.14 ± 0.02 ^c^
C	71.69 ± 0.01 ^b^	−0.93 ± 0.01	19.91 ± 0.02 ^b^

A—powder obtained at 160 °C; B—powder obtained at 180 °C; and C —powder obtained at 200 °C. Values are means ± standard deviation of triplicate determinations. Means with different letters in the same column are significantly different at *p* < 0.05.

**Table 5:** D (_4_,_3_) and SPAN values of emulsions prepared with various content of FOCE powders.

Emulsion	D_4.3_ (µm)	SPAN (-)
A—0.5%	37.42 ± 0.20 ^a^	8.02
B—0.5%	22.52 ± 0.21 ^b^	6.27
C—0.5%	20.91 ± 0.05 ^d^	5.22
A—1%	21.56 ± 0.05 ^c^	7.20
B—1%	18.76 ± 0.10 ^e^	5.53
C—1%	8.12 ± 0.10 ^f^	4.39
A—3%	7.89 ± 0.11 ^g^	6.76
B—3%	7.86 ± 0.23 ^h^	4.40
C—3%	7.51 ± 0.02 ^i^	3.71

A—emulsions prepared with powder obtained at 160 °C; B—emulsions prepared with powder obtained at 180 °C; C—emulsions prepared with powder obtained at 200 °C; and 0.5%, 1%, and 3%—powder contents. Values are means ± standard deviation of triplicate determinations. Means with different letters in the same column are significantly different at *p* < 0.05.

**Table 6:** Color values of emulsions prepared with various content of FOCE powders.

Emulsion	L*	a*	b*
A—0.5%	86.36 ± 0.05 ^f^	−1.94 ± 0.01 ^i^	12.29 ± 0.05 ^h^
A—1%	86.42 ± 0.02 ^f^	−1.35 ± 0.01 ^e^	15.99 ± 0.02 ^e^
A—3%	83.41 ± 0.00 ^g^	−0.59 ± 0.00 ^a^	18.80 ± 0.01 ^a^
B—0.5%	88.57 ± 0.02 ^a^	−1.61 ± 0.01 ^f^	17.31 ± 0.02 ^b^
B—1%	87.18 ± 0.04 ^e^	−1.64 ± 0.00 ^g^	16.94 ± 0.04 ^c^
B—3%	88.49 ± 0.02 ^b^	−1.21 ± 0.00 ^d^	16.81 ± 0.01 ^d^
C—0.5%	88.57 ± 0.02 ^a^	−1.80 ± 0.00 ^h^	12.93 ± 0.00 ^g^
C—1%	87.33 ± 0.02 ^d^	−1.12 ± 0.01 ^c^	15.78 ± 0.01 ^f^
C—3%	87.64 ± 0.02 ^c^	−0.78 ± 0.01 ^b^	15.98 ± 0.01 ^e^

A—emulsions prepared with powder obtained at 160 °C; B—emulsions prepared with powder obtained at 180 °C; C—emulsions prepared with powder obtained at 200 °C; and 0.5%, 1%, and 3%—powder contents. Values are means ± standard deviation of triplicate determinations. Means with different letters in the same column are significantly different at *p* < 0.05.
